# AAV9-mediated Schwann cell-targeted gene therapy rescues a model of demyelinating neuropathy

**DOI:** 10.1038/s41434-021-00250-0

**Published:** 2021-03-10

**Authors:** Alexia Kagiava, Christos Karaiskos, Jan Richter, Christina Tryfonos, Matthew J. Jennings, Amanda J. Heslegrave, Irene Sargiannidou, Marina Stavrou, Henrik Zetterberg, Mary M. Reilly, Christina Christodoulou, Rita Horvath, Kleopas A. Kleopa

**Affiliations:** 1 Neuroscience Department, The Cyprus Institute of Neurology and Genetics and Cyprus School of Molecular Medicine, Nicosia, Cyprus; 2 Department of Molecular Virology, The Cyprus Institute of Neurology and Genetics and Cyprus School of Molecular Medicine, Nicosia, Cyprus; 3 Department of Clinical Neurosciences, University of Cambridge, Cambridge, United Kingdom; 4 Department of Neuromuscular Diseases, UCL Queen Square Institute of Neurology, London, United Kingdom; 5 Department of Neurodegenerative Disease, UCL Institute of Neurology, London, United Kingdom; 6 UK Dementia Research Institute at UCL, London, United Kingdom; 7 Department of Psychiatry and Neurochemistry, Institute of Neuroscience and Physiology, the Sahlgrenska Academy at the University of Gothenburg, Mölndal, Sweden; 8 Clinical Neurochemistry Laboratory, Sahlgrenska University Hospital, Mölndal, Sweden; 9 Center for Neuromuscular Disorders, The Cyprus Institute of Neurology and Genetics and Cyprus School of Molecular Medicine, Nicosia, Cyprus

**Keywords:** Peripheral nervous system, Neurological disorders, Myelin biology and repair, Biomarkers

## Abstract

Mutations in the *GJB1* gene, encoding the gap junction (GJ) protein connexin32 (Cx32), cause X-linked Charcot-Marie-Tooth disease (CMT1X), an inherited demyelinating neuropathy. We developed a gene therapy approach for CMT1X using an AAV9 vector to deliver the *GJB1/Cx32* gene under the myelin protein zero (*Mpz*) promoter for targeted expression in Schwann cells. Lumbar intrathecal injection of the AAV9*-Mpz.GJB1* resulted in widespread biodistribution in the peripheral nervous system including lumbar roots, sciatic and femoral nerves, as well as in Cx32 expression in the paranodal non-compact myelin areas of myelinated fibers. A pre-, as well as post-onset treatment trial in *Gjb1*-null mice, demonstrated improved motor performance and sciatic nerve conduction velocities along with improved myelination and reduced inflammation in peripheral nerve tissues. Blood biomarker levels were also significantly ameliorated in treated mice. This study provides evidence that a clinically translatable AAV9-mediated gene therapy approach targeting Schwann cells could potentially treat CMT1X.

## Introduction

X-linked Charcot-Marie-Tooth (CMT1X) is the second most common inherited demyelinating neuropathy [1] characterized by slowly progressive muscle weakness and atrophy, loss of reflexes, and reduced nerve conduction velocities [2–5]. CMT1X affects mostly men with onset at the age of 5–20 years [5–7], while affected women may present at a later age [8, 9] with milder symptoms [10].

CMT1X is caused by mutations in the *GJB1* gene encoding the gap junction (GJ) protein connexin 32 (Cx32). Cx32 is a myelin-related protein that forms intracellular GJ channels through the non-compact myelin layers at paranodal loops and Schmidt–Lantermann incisures of myelinating Schwann cells [11–13] serving important homeostatic and axon-glial signaling functions. There are over 400 different *GJB1* mutations reported so far (http://hihg.med.miami.edu/code/http/cmt/public_html/index.html#/) affecting all domains of Cx32, as well as the non-coding gene regions [14, 15]. Frameshift, premature stop, and non-coding mutations are predicted to cause complete loss of protein function through failed synthesis or rapid degradation. Several missense and inframe mutations are retained intracellularly [16–18] in the ER and/or Golgi [18–22] with the inability to form functional channels, while other mutants form membrane channels with altered biophysical characteristics [22].

Despite the large number and different mechanisms of CMT1X-associated mutations, clinical studies have demonstrated that males suffering from the disease have a similar phenotype and severity that correlates with age and is independent of the specific mutation, comparable with the phenotype of patients with a complete *GJB1* deletion [23, 24]. Furthermore, *Gjb1*-null mice with deletion of the *Gjb1/Cx32* gene develop a progressive, predominantly motor demyelinating peripheral neuropathy beginning at about three months of age with reduced sciatic motor nerve conduction velocity (MNCV) and motor amplitude [25, 26]. Expression of wild type (WT) human Cx32 protein driven by the rat myelin protein zero promoter (*Mpz*/P0) promoter prevented demyelination in *Gjb1*-null mice [27], confirming that loss of Schwann cell autonomous expression of Cx32 is sufficient to cause CMT1X pathology. Transgenic mice expressing CMT1X mutations showed no detectable Cx32 protein in the 175 fs mutant line [28], while R142W, T55I, R75W and N175D transgenic mice showed retention of the mutant protein in the perinuclear region and developed a demyelinating neuropathy similar to Cx32 KO mice [29, 30]. Taken together, these findings suggest that most *GJB1* mutations cause Schwann cell-autonomous loss of Cx32 function, indicating that gene replacement therapy is a valid approach to treat the disease.

We previously showed that a single lumbar intrathecal injection of a lentiviral vector driven by the *Mpz* promoter carrying the *GJB1* gene can partially improve the demyelinating neuropathy in *Gjb1*-null mice both at early and at later stages of the disease [31, 32]. However, low levels of lentiviral derived expression may limit the potential for translating this approach for a subset of CMT1X patients with Golgi-retained interfering mutations [33, 34]. In addition, the mutagenic risk of lentiviral vectors through genome integration [35] limits their potential to be used in the treatment of patients. Therefore, we considered as an alternative approach the use of an adeno-associated viral vector (AAV). AAV vectors have been extensively used in the last decades for gene delivery mainly to the central nervous system (CNS) and in some applications also to the peripheral nervous system (PNS) [36–39]. Although previous studies showed that the genes carried by AAV vectors can be expressed in the PNS, expression was driven by ubiquitous promoters and was not targeted to Schwann cells. Thus, the improvement observed could result from cross-correction through the transfer of the protein from axons and other cells surrounding the PNS. The *Mpz* promoter has been used to target Schwannoma cells with AAV1 vectors resulting in the regression of the tumors following intraneural injection [40, 41].

In order to develop translatable gene therapy for CMT1X, we used here an AAV9 vector driven by the Schwann cell-specific *Mpz* promoter in order to achieve targeted *GJB1* gene expression in Schwann cells throughout the PNS. Following a single lumbar intrathecal injection, we demonstrate widespread vector biodistribution to the lumbar roots, as well as to sciatic and femoral nerves, leading to high expression rates of Cx32 specifically in myelinating Schwann cells. Using this approach, we demonstrate a significant therapeutic benefit in the CMT1X mouse model when treated before as well as after the onset of peripheral neuropathy. This study provides the proof of principle for a gene therapy approach to treat CMT1X patients.

## Materials and methods

### Cloning and production of AAV vectors

**Fig. 1:**
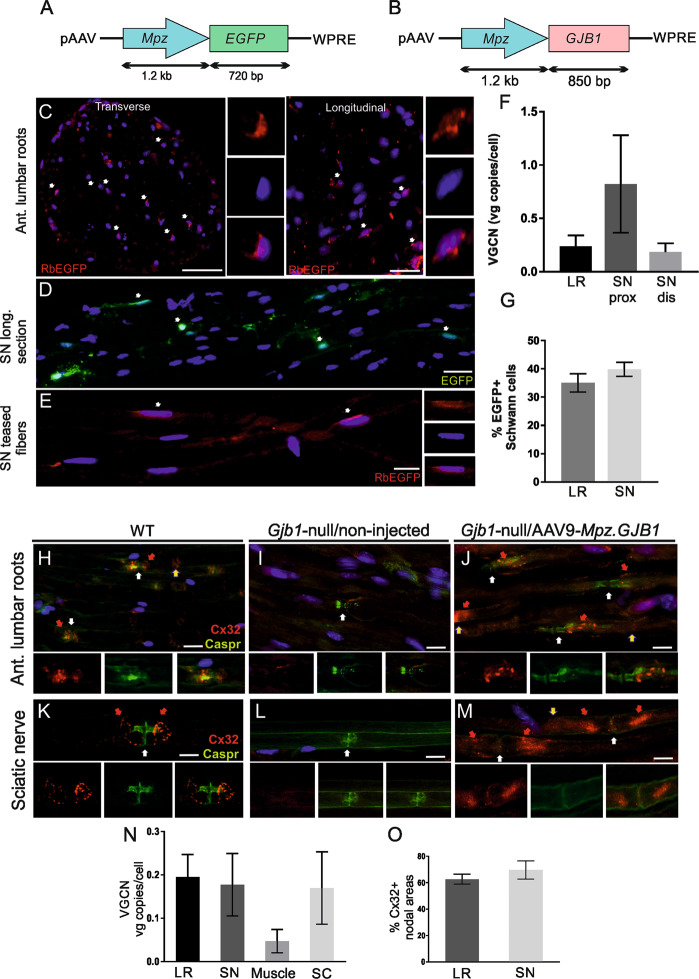
Biodistribution and expression of AAV9-*Mpz*.*Egfp* and AAV9-*Mpz*.*GJB1* vectors. Diagrams showing the expression cassette of the AAV9-*Mpz*.*Egfp* (mock) (**A**) and AAV9-*Mpz*.*GJB1* (full) (**B**) vectors, in which the expression of either the EGFP reporter gene or of the human *GJB1* open reading frame, respectively, is driven by the rat myelin protein zero (*Mpz*) promoter. Immunostaining for EGFP (red) of lumbar root sections (**C**) and sciatic nerve teased fibers (**E**) reveals perinuclear EGFP immunoreactivity (arrows) in a subset of Schwann cells 8 weeks after intrathecal injection of AAV9-*Mpz*.*Egfp* vector in wild type (WT) mice. Auto-fluorescent EGFP signal (green) is also evident in sciatic nerve sections without antibody detection (**D**). Cell nuclei are stained with DAPI (blue). Determination of the vector genome copy numbers (VGCN) confirms AAV9 biodistribution to lumbar roots (LR) as well as both proximal (prox) and distal (dis) sciatic nerves (SN) (**F**). Quantification of the percentage of EGFP-positive cells in lumbar roots and sciatic nerves shows no significant differences between PNS tissues examined at 8 weeks post-injection (**G**). Following injection of AAV9-*Mpz*.*GJB1* in *Gjb1*-null mice immunostaining of lumbar root sections (**H**)–(**J**) and sciatic nerve teased fibers (**K**)–(**M**) for Cx32 (red) and paranodal axonal marker Caspr (white arrows) shows that virally delivered Cx32 (red arrows) is correctly expressed at some of the paranodal myelin areas (white arrows) only in injected *Gjb1*-null mice (**J**) and (**M**) similar to WT mice (**H**) and (**K**) but not in their non-injected *Gjb1*-null littermates (**I**) and (**L**). Cx32 is also detected in the Schmidt-Lanterman incisures (yellow arrows) in both WT (**A**) and AAV9-*Mpz*.*GJB1* injected mice (**J**) and (**M**). **N** VGCN determination demonstrates biodistribution of AAV9-*Mpz*.*GJB1* to both lumbar roots and sciatic nerves in addition to the spinal cord (SC) and to a lesser degree to the quadriceps muscle. **O** Quantification of the percentage of Cx32-positive paranodal areas in lumbar roots and sciatic nerves shows comparable results in both tissues. VGCNs and expression rates values represent Mean ± SEM. Scale bars: (**C**) and (**D**): 20 μm; (**E**)–(**M**): 10 μm.

We generated novel constructs for AAV vector gene delivery designed to provide Schwann cell-specific expression of either the reporter gene EGFP (pAAV-*Mpz.Egfp*, mock vector; Fig. 1A) or Cx32 (pAAV-*Mpz.GJB1*, full, therapeutic vector; Fig. 1B), both under the rat 1.2 kB *Mpz* promoter shown to drive expression specifically in Schwann cells [27, 32]. The *Mpz*/Cx32 ORF was PCR amplified from a lentiviral construct previously made in the lab [42]. The primers used for the amplification were P0-Cx32-F 5′–AGGGGTACCCTTCCTGTTCAGACT–3′ (SEQ ID No. 13) and P0-Cx32- R 5′–CCGCTCGAGGGATCCTC AGCAG–3′. The PCR product (2030 bp) was gel purified and digested using KpnI and XhoI. The AAV vector was also digested with the same restriction enzymes. The entire expression cassette was confirmed by direct sequencing of the ORFs.

The pAAV-*Mpz.Egfp* and pAAV-*Mpz.GJB1* plasmids were cross-packaged into AAV9 capsid. For AAV9 vector production HEK293T, cells are grown in 10 cm tissue culture dishes in IMDM supplemented with 10% FCS. Two hours prior to transfection, the medium is changed. When cells have reached ~70–80% confluency, they are transfected using polyethylenimine (PEI; branched, MW 25,000; Sigma) with a three-plasmid mix of pAAV, pRepCap, and pXX6 plasmid provided by Dr. Jude Samulsky, the University of North Carolina at Chapel Hill at a ratio of 1:2:1 and the medium is changed 16 h after transfection. As it was shown previously [43] that the cell media contains significant amounts of virions, both the cells and the cell supernatant are collected 48 h later for vector purification. Cells are pelleted by centrifugation at 500 g for 10 min and are subsequently re-dissolved in lysis buffer (0.5% sodium deoxycholate, 150 mM NaCl, 20 mM Tris, 50 U/ml Benzonase; Sigma-Aldrich, Munich, Germany) and incubated at 37 °C for 1 h. The lysate is then clarified by centrifugation at 3000 g for 10 min and filtered in parallel with the supernatant through 0.45 µm Millipore filters (Millipore, San Salvador, El Salvador). Next, RNAseA and a Protease inhibitor cocktail are added to both the lysate and the supernatant and incubated for 2 h at 37 °C followed by clarification at 3000 g for 15 min. The preparations are then combined with a precipitation mix (PEG/NaCl) at a ratio of 3:1, which is incubated at 4 °C overnight. The mixture is centrifuged at 3000 g for 30 min and the aqueous supernatant discarded. The virion-containing pellet is then resuspended in 3 ml Pellet suspension buffer (250 mM NaCl solution), which is clarified by centrifugation at 10,000 g for 10 min at 4 °C. The virus-containing aqueous layer is then transferred to the Beckmann UltraClear SW41 tube and is centrifuged at 149,000 g for 3 h. The aqueous layer is then discarded and the viral pellet resuspended in 200 µl pellet suspension buffer. The vector genome copy number (VGCN) is determined by qPCR targeting the WPRE sequence [44].

### Experimental animals

In this study, we used adult WT C57BL/6 or *Gjb1-*null/Cx32 KO (C57BL/6_129) mice weighing 20–25 g. Early and late gene therapy trials were conducted using 2- and 6-month-old mice *Gjb1-*null/Cx32 KO mice weighing 20–25 g. *Gjb1-*null/Cx32 KO mice were obtained from the European Mouse Mutant Archive, originally generated by Prof. Klaus Willecke (University of Bonn). In these mice, the *neor* gene was inserted in-frame into the exon 2 of *Gjb1* gene which contains the ORF [45]. Mice were kept in a specific pathogen-free animal facility, housed in open-top system cages. Wood bedding for laboratory mice, dried by high-temperature treatment, sieved, de-dusted, of high absorbency, was used and mice were housed up to five in each cage. Standard mouse diet, certificate, for reproduction, weaning, growth, and tap potable water, filtered and UV sterilized were administered to the mice. Mice were kept in a 12 h dark/12 h light cycle at a temperature of 22 °C. Both male and female mice were used in our experiments and showed no (sex-related) differences in their behavioral performance or nerve pathology. All experimental procedures in this study were conducted in accordance with animal care protocols approved by the Cyprus Government’s Chief Veterinary Officer (project license CY/EXP/PR.L3/2017) according to national law, which is harmonized with EU guidelines (EC Directive 86/609/EEC).

### Study design

For the analysis of EGFP expression, we used 6 WT mice injected with the pAAV-*Mpz.Egfp* (mock) vector and for Cx32 expression analysis, we used ten *Gjb1-*null mice injected with the pAAV-*Mpz.GJB1* (full) vector. For the early treatment study, we used ten *Gjb1-*null mice injected with the mock vector and 10 injected with the full vector. Finally, for the late treatment 20 *Gjb1-*null mice were used in the mock group and 20 in the full group. Untreated *Gjb1-*null mice (*n* = 7), as well as WT mice (*n* = 4) of the same age, were also assessed for the same outcome measures. We used 3–4 mice in each group for the expression analysis in order to obtain reliable data for statistical analysis (*t*-test). For the treatment trials, we used 8–12 mice for both electrophysiological and morphological analysis in order to overcome the variability between mice and obtain reliable data for statistical analysis. We did not exclude outliers in case we had. Foot grip tests were performed three times and the mean value was used while muscle contraction experiments were performed twice.

**Fig. 2:**
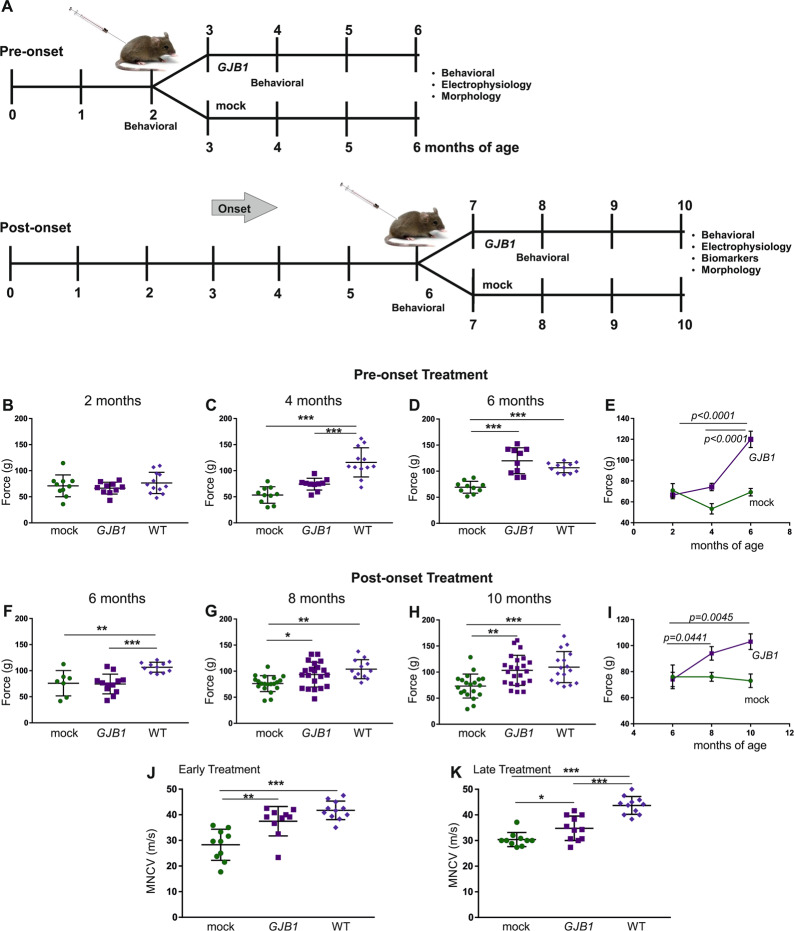
Analysis of functional outcomes of AAV9-*Mpz*.*GJB1* injected *Gjb1*-null mice compared to AAV9-*Mpz*.*Egfp* (mock) treated littermates. **A** Diagram showing the pre- and post-onset treatment trial time course with a timing of functional and morphological analysis. Results of foot grip analysis in pre- (**B**)–(**E**) and post-onset treatment groups (**F**)–(**I**) comparing AAV9-*Mpz.GJB1* treated (*GJB1*) and mock-treated *Gjb1*-null mice, as indicated. There were no differences between the treatment groups before the injection (**B**) and (**F**), while 2 months post-injection grip strength improved in both pre- and post-onset treated *Gjb1*-null mice compared to the mock groups (*n* = 10 per group) (**C**) and (**G**). The improved performance continued at 4 months post-injection in both pre- (6-month old) and post-onset (10-months old) treated mice compared to controls and was not significantly different from the performance of wild type (WT) mice of the same age (**D**) and (**H**). Longitudinal analysis within each group showed improved motor performance over time in both pre- and post-onset treated *Gjb1*-null mice while mock groups did not improve (**E**) and (**I**). Sciatic nerve motor conduction velocity (MNCV) also showed significant improvement 4 months after treatment compared to the mock groups in both pre- (**J**) and post-onset (**K**) treated mice (*n* = 10 for all groups). MNCVs in early treated mice did not differ significantly from those of age-matched WT mice (**J**) while in post-onset treated mice they remained below those of age-matched WT mice (**K**) (One-way ANOVA with Bonferroni post-test; *P < 0.05, **P < 0.01 and ***P < 0.001).

The aim of this study was to examine whether a gene addition therapy can treat peripheral neuropathy or prevent the development of peripheral neuropathy in the mouse model of CMT1X neuropathy both at early and late stages, before and after the onset of the neuropathy. The gene therapy study was conducted using four groups of *Gjb1*-null mice. A minimum of 8–12 mice per treatment group for each outcome measure was considered adequate for assessing statistically significant differences based on our previous studies using similar models [32, 42]. Mice were treated at the age of 2 months for the early treatment and at 6 months of age for the late treatment (Fig. 2A). Littermate mice were randomized to receive either AAV9-*Mpz*.*GJB1* (full) treatment or AAV9-*Mpz*.*Egfp* (mock treatment, control group) and were assigned a coding number for further identification. 2-month-old mice were evaluated before the treatment, and again at the age of 4 and 6 months, while 6-month-old mice were evaluated before the treatment, and at the age of 8 and 10 months. Mice were evaluated by behavioral testing by an examiner blinded to the treatment condition and used at the age of 6 months for the early treatment and 10 months for the late treatment for electrophysiology or for quantitative morphometric analysis of semi-thin sections. Animals subjected to muscle contraction experiments were not used for morphological analysis. Analysis of physiological and morphological results was also performed blinded to the treatment condition.

### Intrathecal vector delivery

We delivered the AAV vectors by a single lumbar intrathecal injection as previously described [32, 46]. Briefly, a small skin incision was made along the lower lumbar spine level of anesthetized mice to visualize the spine and the AAV vector was delivered into the L5-L6 intervertebral space. A 50-μL Hamilton syringe (Hamilton, Giarmata, Romania) connected to a 26-gauge needle was used to inject 20 µL of AAV stock containing an estimated 2 × 10^10^ vector genomes (vg)/mL, at a maximum rate of 5 µL/min. A flick of the tail was considered indicative of successful intrathecal administration.

### VGCN determination

Genomic DNA was extracted from different PNS tissues (i.e., lumbar roots, proximal and distal sciatic nerves, and femoral motor nerves) of mice 4 or 8 weeks after intrathecal vector delivery using the Invitrogen iPrep PureLink gDNA Kit (Thermo Fisher Scientific, Waltham, MA USA). The extracted DNA was analyzed for yield and purity using a Nanodrop 1000 spectrophotometer. Approximately 20 ng of DNA was used as template for two real-time PCR assays on an Applied Biosystems 7500 Real-Time PCR System involving 45 cycles of 15 s at 95 °C and 60 s at 60 °C. TFRC-specific primers/probe targeting the mouse genome and WPRE-specific primers/probe, which is contained in the transgene, were used. Standard curves were created by serial dilution of quantified mouse genomic DNA, as well as quantified plasmid DNA containing the transgene cassette. The average VCN per cell was calculated as the total VCN divided by the total cell number.

### Immunofluorescence staining

For immunostaining, mice were anesthetized with avertin according to institutionally approved protocols, and then transcardially perfused with normal saline followed by fresh 4% paraformaldehyde in 0.1 M PB buffer. The lumbar-sacral spinal cords with spinal roots attached, as well as the bilateral sciatic and femoral motor nerves were dissected and post-fixed in 4% PFA, the spinal cord for 2 h while sciatic and femoral nerves for 30 min. Spinal roots were frozen for cryosections while sciatic and femoral nerves were isolated and teased into fibers under a stereoscope. Teased fibers or sections were permeabilized in cold acetone and incubated at RT with a blocking solution of 5% BSA (Sigma-Aldrich, Munich, Germany) containing 0.5% Triton-X (Sigma-Aldrich, Munich, Germany) for 1 h. Primary antibodies used were: mouse monoclonal antibody against the contactin-associated protein (Caspr, 1:50; gift of Dr. Elior Peles, Weizmann Institute of Science), NeuN (1:400 Chemicon, San Salvador, El Salvador), CC1 (1:50, Calbiochem, San Salvador, El Salvador), GFAP (1:400, Sigma-Aldrich, Munich, Germany), rabbit antisera against EGFP (1:1,000; Invitrogen, Waltham, MA USA), Caspr2 (1:200, Alomone Labs, Jerusalem, Israel), CD3 (1:100; Abcam, Cambridge, UK) and Cx32 (1:50; Invitrogen, Waltham, MA USA), rat CD68 (1:50; Biorad, California, USA), CD45 (1:100; Abcam, Cambridge, UK) and goat CD20 (1:100; Santa Cruz, Texas, USA) all diluted in blocking solution and incubated overnight at 4 °C. Slides were then washed in PBS and incubated with mouse cross-affinity fluorescein-conjugated (1:1000; Invitrogen, Waltham, MA USA), rat cross-affinity purified rhodamine-conjugated (1:2000; Invitrogen, Waltham, MA USA), goat cross-affinity fluorescein-conjugated (1:700; Abcam, Cambridge, UK) and rabbit cross-affinity purified rhodamine-conjugated (1:500; Jackson ImmunoResearch, West Grove, USA) secondary antibodies for 1 h at RT. Cell nuclei were visualized with DAPI (1 µg/ml; Sigma, Munich, Germany). Slides were mounted with fluorescent mounting medium and images photographed under a fluorescence microscope with a digital camera using a fluorescence microscope (Nikon Eclipse N*ἱ*; Tokyo, Japan) with a digital camera (DS-Qi2) using NIS-Elements software.

### Immunoblot analysis

Fresh sciatic and femoral nerves and lumbar spinal roots were collected at 8 weeks post-injection and lysed in ice-cold RIPA buffer (10 mM sodium phosphate, pH 7.0, 150 mM NaCl, 2 mM EDTA, 50 mM sodium fluoride, 1% Nonidet P-40, 1% sodium deoxycholate, and 0.1% SDS all from Sigma-Aldrich, Munich, Germany) containing a mixture of protease inhibitors (Roche, Sigma-Aldrich, Munich, Germany). Proteins (150 μg) from the lysates were fractionated by 12% SDS/PAGE and then transferred to a Hybond-C Extra membrane (GE Healthcare Life Sciences, Logan, USA) using a semidry transfer unit. Nonspecific sites on the membrane were blocked with 5% non-fat milk in PBS with Tween 20 (PBST) for 1 h at room temperature. Immunoblots were incubated with rabbit antisera against EGFP (1:1000; Abcam, Cambridge, UK) or Cx32 (clone 918, 1:3000) [47] and mouse β-tubulin (1:4000; Developmental Studies Hybridoma Bank, Iowa, USA) at 4 °C overnight. After washing, the immunoblots were incubated with an anti-mouse or anti-rabbit HRP-conjugated secondary antiserum (Jackson ImmunoResearch, diluted 1:3000, West Grove, USA) in 5% milk–PBST for 1 h. The bound antibody was visualized by an enhanced chemiluminescence system (GE Healthcare Life Sciences, Logan, USA).

### Behavioral analysis

#### Grip strength testing

To measure grip strength, mice were held by the tail and lowered towards the apparatus (Ugo Basile, Varese, Italy) until they grabbed the grid with the hind paws. Mice were gently pulled back until they released the grid. Measurements of the force in g were indicated on the equipment. Each session consisted of three consecutive trials and measurements were averaged. Hind limb force was compared between AAV9.*Mpz*-*GJB1* and AAV9.*Mpz*-*Egfp* treated mice.

### Electrophysiological analysis

#### Motor nerve conduction velocity (MNCV)

MNCV was measured in vivo using published methods [48] from bilateral sciatic nerves following stimulation in anesthetized animals using two stimulation sites, one near the sciatic notch and one distally at the ankle via bipolar electrodes with supramaximal square-wave pulses (5 V) of 0.05 ms. The latencies of the compound muscle action potentials (CMAP) were recorded by a bipolar electrode inserted between digits 2 and 3 of the hind paw and measured from the stimulus artifact to the onset of the negative M-wave deflection. MNCV was calculated by dividing the distance between the stimulating and recording electrodes by the result of subtracting distal from proximal latency.

#### Quadriceps muscle contractility study

Quadriceps muscle contractility study was performed only in the late treatment group in order to assess in situ the function of the lumbar root and femoral motor axons. Mice subjected to muscle contraction study were not used for morphological assessment. We measured the contraction properties of the quadriceps muscle innervated by the femoral nerve in an anesthetized mouse as previously described [32]. After exposure of the motor part of the femoral nerve a stimulating hook electrode was used to stimulate the motor branch of the femoral nerve at 1 Hz using a constant current stimulator (DS3; Digitimer, Welwyn Garden City, UK) with 5–6 mA and 200 μs duration pulse. The muscle contraction of the partially exposed quadriceps muscle was recorded with a force displacement transducer (FT03; Grass Technologies), which was attached to the muscle with a silk suture. The transducer was connected to a micromanipulator and for the experiment the muscle was extended 1 mm each time until the muscle contraction reached the maximum value. The average amplitude and duration of the force generated by the quadriceps muscle contraction was compared between treatment groups.

### Biomarkers determination in blood samples of *Gjb1*-null treated mice

In order to further access the potential value of clinically relevant blood biomarkers and their treatment responsiveness, we performed biomarker analysis from blood samples of WT compared to untreated *Gjb1*-null mice, as well as from *Gjb1*-null mice treated either with the therapeutic or with the mock vector, all at the age of 10 months. Blood was collected prior to sacrificing the animals using standard methods [49]. We measured the levels of neurofilament light (NF-L) and neural cell adhesion molecule (NCAM-1).

#### Measurement of NF-L concentration

Blood samples were processed within one hour. Blood was collected into EDTA-containing tubes and centrifuged at 20 °C at 3500 rpm for 10 min. Plasma was aliquoted and stored at −80 °C until testing. Plasma NF-L concentration was measured at University College London (UCL) using a commercially available NF-Light kit on a Single molecule array (Simoa) HD-1 instrument (Quanterix, Billerica, MA) [50, 51].

#### Measurement of NCAM-1 concentration by ELISA

Serum was separated using 0.5 ml Minicollect Z serum separation tubes. Blood samples were taken and centrifuged 20 °C at 3000 g for 10 min. Serum was aliquoted and stored at −80 °C until testing. Serum protein levels were determined at the Department of Clinical Neurosciences, University of Cambridge, by ELISA according to manufacturers’ instructions for neural cellular adhesion molecule (NCAM1; Rockland KOA0716).

### Morphometric analysis of myelination in lumbar roots and peripheral nerves

Mice were transcardially perfused with 2.5% glutaraldehyde in 0.1 M PB buffer. The lumbar spinal cord with multiple spinal roots attached, as well as the femoral and sciatic nerves, were dissected and fixed overnight at 4 °C, then osmicated, dehydrated, and embedded in araldite resin (all purchased from Agar Scientific, Essex, UK). Transverse semi-thin sections (1 μm) of the lumbar spinal cord with roots and the middle portion of the femoral motor and sciatic nerves were obtained and stained with alkaline toluidine blue (Sigma-Aldrich, Munich, Germany). Sections were visualized with 10×, 20×, and 40× objective lenses and captured using a Nikon Eclipse Ni microscope (Tokyo, Japan) with a digital camera (DS-Fi3) using NIS-Elements software. Images of whole root or transverse nerve sections were obtained at 100–200× final magnification, and a series of partially overlapping fields covering the entire cross-sectional area of the roots or the nerves were captured at 400× final magnification.

These images were used to examine the degree of abnormal myelination in all treatment groups as described previously [30, 42, 52]. In brief, all demyelinated, remyelinated, and normally myelinated axons were counted in the entire root or nerve cross-section using the following criteria: axons larger than 1 μm without a myelin sheath were considered demyelinated; axons with myelin sheaths <10% of the axonal diameter and/or axons surrounded by “onion bulbs” (i.e., circumferentially arranged Schwann cell processes and extracellular matrix) were considered remyelinated; all other myelinated axons were considered normally myelinated.

In addition, we counted the number of foamy macrophages present within the entire cross section of each root or nerve, as an indication of inflammation. Macrophages were identified in semi-thin sections at 400× magnification as cells laden with myelin debris, devoid of a basement membrane, and extending small, microvilli-like processes, as described previously [53, 54]. The macrophage count was calculated as the ratio per 1000 myelinated fibers, to account for size differences between different spinal roots and nerves. All pathological analyses were performed blinded to the treatment condition of each mouse.

### Statistical analysis

The percentages of EGFP-positive Schwann cells or Cx32-expressing paranodal myelin areas in immunostained spinal roots and sciatic nerves of WT and *Gjb1*-null mice injected with the mock or full vector, respectively, were compared with Student’s *t*-test. Behavioral testing results, electrophysiological results, and blood biomarker levels were compared using one-way ANOVA with Bonferroni post-test. Morphological analysis data obtained from mock-treated and fully treated groups were compared using the Mann–Whitney *U* test (significance level for all comparisons, *P* < 0.05). All tests were performed using GraphPad Instat3 software (GraphPad, San Diego, USA).

## Results

### EGFP expression following intrathecal injection of AAV9-*Mpz*.*Egfp*

AAV9 vectors (both mock and full) were produced to titers of 1 × 10^12^ vg/ml. AAV9-*Mpz*.*Egfp* (mock vector; Fig. 1A) was delivered by lumbar intrathecal injection (estimated 2 × 10^10^ vg injected) into WT mice in order to study the biodistribution of the vector throughout the PNS. VGCNs and EGFP expression were examined 8-weeks post-injection in anterior lumbar roots and sciatic nerves. EGFP was detected by immunofluorescent labeling (Fig. 1C, E) as well as by auto-fluorescence without the use of an antibody (Fig. 1D) in the perinuclear cytoplasm of a subset of Schwann cells in both the lumbar roots and sciatic nerves. VGCNs in DNA extracted from PNS tissues reached 0.24 ± 0.09 in anterior lumbar roots, 0.82 ± 0.46 in the proximal part of the sciatic nerve and 0.18 ± 0.08 in the distal part of the sciatic nerve (*n* = 4 mice; Fig. 1F). EGFP expression rates in immunostained tissue sections reached 35.0 ± 3.23 % in anterior lumbar roots and 39.9 ± 2.49 % in sciatic nerves (*n* = 3 mice; Fig. 1G). EGFP was also detected by immunoblot analysis of lumbar roots, sciatic and femoral nerve samples of injected mice with a proximal to distal expression level gradient, while it was not detected in non-injected WT mouse samples run as negative controls (Fig. S1A–C).

In order to confirm the cell-specificity of the Mpz promoter we performed double immunostaining for EGFP with cell markers in lumbar spinal cord sections and confirmed that EGFP expression was not detected in oligodendrocytes, neurons, or astrocytes (Fig. S2A–C). Likewise, staining of sciatic nerve sections with vimentin, a fibroblast marker, and Glut-1, a perineurial cell marker, showed no EGFP expression either in fibroblasts or in perineurial cells (Fig. S2D, E). Furthermore, in order to exclude an inflammatory response that could occur after intrathecal injection of the vector we immunostained sections of spinal cord with anterior lumbar roots attached using different inflammatory cell markers. We did not detect any CD68^+^ macrophages, CD3^+^ T lymphocytes, CD45^+^ leukocytes or CD20^+^ B-lymphocytes in CNS or PNS tissues of injected mice (Fig. S3).

### Cx32 expression following intrathecal injection of AAV9-*Mpz*.*GJB1*

AAV9-*Mpz*.*GJB1* (full, therapeutic vector; Fig. 1B) driving expression of the human *GJB1* open reading frame was delivered by lumbar intrathecal injection (estimated 2 × 10^10^ vg injected) into both 2- and 6-month-old *Gjb1*-null mice in order to study the expression of Cx32 throughout the PNS in the absence of endogenous Cx32 at the age groups relevant for the treatment trials. Cx32 expression and VGCNs were determined 4 weeks post-injection in anterior lumbar roots and sciatic nerves. Cx32 was detected as expected at the paranodal areas of myelinating Schwann cells in both lumbar roots and sciatic nerves, similar to the WT tissues (Fig. 1H–M). At 4 weeks post-injection, VGCNs reached 0.2 ± 0.05 (*n* = 4 mice) in anterior lumbar roots and 0.18 ± 0.07 in the sciatic nerve (*n* = 4 mice; Fig. 1N). We also measured the VGCNs in other tissues including the quadriceps muscles (0.05 ± 0.03), the spinal cord (0.17 ± 0.08), and the liver (17.32 ± 3.30). The percentage of Cx32-immunoreactive paranodal areas among all paranodal areas visualized in each tissue based on Caspr paranodal labeling was quantified reaching 62.7 ± 3.75 % in anterior lumbar roots (*n* = 5 mice) and 69.9 ± 6.90 % in sciatic nerves (*n* = 5 mice) (Fig. 1O). Immunoblot analysis confirmed high expression levels of Cx32 in anterior lumbar root and sciatic nerve lysates from injected animals similar to animals transgenically expressing Cx32, while Cx32 was absent in non-injected *Gjb1*-null mice tissues (Fig. S1D, C).

### Improvement of motor performance in pre- and post-onset treated *Gjb1*-null mice

Following confirmation of therapeutic vector expression in both 2- and 6-month-old *Gjb1*-null mice, we proceeded with pre- and post-onset treatment trials in which littermate mice were randomized into two groups, one receiving the mock (AAV9-*Mpz.Egfp*) and the other the full (AAV9-*Mpz.GJB1*) vector. Pre-onset treatment groups were injected at 2 months of age and monitored until 6 months of age when outcome was assessed by behavioral, electrophysiological and morphological analysis. Post-onset treatment groups were injected at 6 months of age and outcome was similarly assessed at 10 months of age, with additional biomarker analysis (Fig. 2A).

Motor performance was assessed before the injection and until the end of the observation period by foot grip analysis in all groups (Fig. 2B–E). Foot grip strength in pre-onset groups at the age of 2 months, at baseline before the initiation of the treatment, showed as expected no difference between the two groups or the WT (*p* > 0.05) with values reaching 66.6 ± 3.59 g for the full vector group (*n* = 10), 71.0 ± 6.69 g for the mock vector group (*n* = 10) and 76.6 ± 5.87 g for the WT group (*n* = 12). At the age of 4 months, 2 months post-injection, muscle strength was increased in fully treated mice reaching 74.3 ± 3.57 g (*n* = 10) compared to the mock group (53.4 ± 5.0; *n* = 10; *p* > 0.05) but without reaching the WT values (116.0 ± 8.06 g; *n* = 12; *p* < 0.0001). At 4 months post-injection the force generated by the hindlimbs of the treated mice was increased reaching 120.0 ± 7.87 g (*n* = 10) compared to the mock group (69.3 ± 3.59 g; *n* = 10; *p* < 0.001) and was similar to the WT values (106.6 ± 2.89; *n* = 12; *p* > 0.05). Longitudinal comparison at different age groups of fully treated mice showed that the force generated by the hindlimbs increased significantly over time (*p* < 0.0001), while mock treated mice did not show any differences (*p* > 0.05).

Foot grip analysis in post-onset treatment groups (Fig. 2F, I) showed no differences at baseline with the values of the fully treated group reaching 74.5 ± 5.46 g (*n* = 21) and the mock 76.0 ± 9.14 g (*n* = 20), while both performed worse than WT mice of the same age (106.6 ± 2.89; *n* = 12; *p* < 0.01). At the age of 8 months, 2 months post-injection, we observed an increase in the force generated by the hindlimbs in the fully treated compared to the mock groups reaching 93.5 ± 5.18 g (*n* = 21) and 76.2 ± 3.41 g (*n* = 20; *p* < 0.05), respectively with the fully treated group reaching values similar to those of the WT mice (103.8 ± 5.26; *n* = 12; *p* > 0.05). At the final stage, at 10 months of age, we observed further improvement of the force in fully treated mice (103.4 ± 6.01 g; *n* = 21), compared to the mock group (73.2 ± 5.15 g; *n* = 20; *p* < 0.01) with no significant difference to WT values (109.6 ± 7.46 g; *n* = 16; *p* > 0.05). Longitudinal comparison of the motor performance at different time points within each treatment group showed that fully treated mice improved significantly 2 months post-injection, from 6 to 8 months of age (*p* = 0.0441) and even further from 8 to 10 months of age (*p* = 0.0045), while mock-treated littermates did not show any significant differences over time (*p* > 0.05). Overall, our motor performance findings at different stages of the treatment trials indicate a continuous improvement of motor performance in both pre- and post-onset treatment groups following the delivery of the AAV9-*Mpz.GJB1* vector.

### Improvement of electrophysiological properties of pre- and post-onset AAV9-*Mpz.GJB1* treated *Gjb1*-null mice

In the pre-onset treatment groups, we performed sciatic MNCV studies at the age of 6 months, 4 months after vector injection. MNCV was improved in the fully treated mice compared to the mock group (*n* = 10 mice per group) as indicated by the values that reached 37.5 ± 1.81 m/s in the fully treated group and 28.3 ± 1.92 m/s in the mock group (*p* < 0.01) (Fig. 2J) without significant difference to the WT values at the same age (41.7 ± 1.09; *n* = 11; *p* > 0.05). Although MNCV was improved in the fully treated mice the amplitude of the CMAP was similar between the two groups reaching 4.6 ± 0.56 mV (*n* = 10) in the mock group and 4.1 ± 0.49 mV (*n* = 10) in the full group (*p* > 0.05; Fig. S4A).

In the post-onset treated groups, we performed both MNCV and quadriceps muscle contraction experiments that reflect chronic axonal loss and muscle denervation. Measurements were performed at 10 months of age, 4 months post-injection. MNCV was improved in the fully treated mice (34.8 ± 1.44 m/s; *n* = 11) compared to the mock group (30.4 ± 0.87 m/s; *n* = 10) (*p* < 0.05; Fig. 2K) but remained significantly below the values of age-matched WT mice (43.7 ± 1.05; *n* = 11; *p* < 0.001). As in the early treated groups, we also measured the CMAP amplitudes and found no significant differences between the two groups (*p* > 0.05) with the values reaching 4.1 ± 0.42 mV (*n* = 11; full group) and 3.2 ± 0.35 mV (*n* = 10; mock group; Fig. S4B). We further examined the muscle contractility properties of the quadriceps muscle after stimulation of the femoral motor nerve in situ (Fig. S4C, D) as previously described [32]. Both force and duration were improved in the AAV9-*Mpz.GJB1* treated (*n* = 11) compared to the mock-treated (*n* = 10) group. Amplitudes reached 0.22 ± 0.010 N in the treated group, similar to WT [32], and 0.18 ± 0.008 N in the mock group (*p* = 0.0059). Furthermore, the duration of contraction was significantly increased in the treated group reaching 0.098 ± 0.004 sec compared to 0.084 ± 0.002 s in the mock group (*p* = 0.0015). Overall, our electrophysiological studies with emphasis on motor fibers that are predominantly affected in this CMT1X model [26, 30] showed significant improvement following both pre- as well as post-onset gene addition therapy.

### Establishment of treatment-responsive blood biomarkers in the CMT1X model

**Fig. 3:**
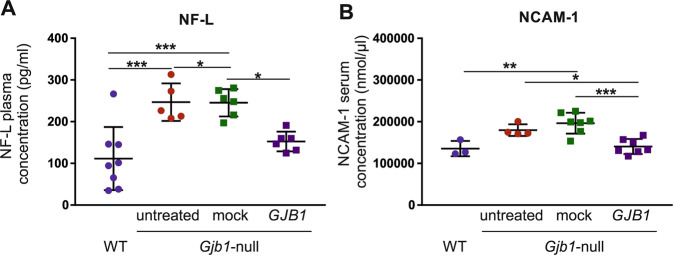
Improvement of blood biomarkers after treatment in *Gjb1*-null mice. Analysis of blood levels of molecules relevant to nerve pathology in 10-month-old AAV9-*Mpz*.*GJB1* treated (*GJB1*) *Gjb1*-null mice compared to AAV9-*Mpz*.*Egfp* (mock) treated littermates, as well as age-matched WT and untreated *Gjb1*-null mice: **A** Neurofilament light (NF-L) plasma concentration is increased in *Gjb1*-null (*n* = 5) compared to WT (*n* = 8) mice and shows significant amelioration in post-onset treated mice compared to mock treated mice (*n* = 6 per group). **B:** Neural cell adhesion molecule-1 (NCAM-1) serum levels were similarly increased in untreated *Gjb1*-null (*n* = 4) compared to WT (*n* = 3) mice and showed significant improvement in treated compared to mock-treated group (*n* = 7 per group) (one-way ANOVA with Bonferroni post-test; *P < 0.05, **P < 0.01 and ***P < 0.001).

Based on previous data showing that biomarkers can be used in order to access treatment outcome in CMT neuropathy models [31, 74] we performed further analysis of blood samples for disease-relevant biomarkers including Neurofilament light (NF-L) and neuronal cell adhesion molecule-1 (NCAM-1). Clinical studies have shown elevation of plasma NF-L levels in patients with different CMT types including CMT1X compared to controls, as well as a correlation between elevated concentrations and disease severity, suggesting that this may be a useful biomarker for assessing disease progression and potential response to treatment in future clinical trials [51]. In keeping with our previous observations [31], we found that plasma NF-L levels were increased in untreated 10-month old *Gjb1*-null mice (246.84 ± 20.18 pg/ml) compared to age-matched WT mice (111.48 ± 26.77 pg/ml; *p* < 0.001). Moreover, comparison of treatment groups (*n* = 6 mice per group) showed that NF-L levels were significantly ameliorated in *Gjb1*-null mice treated post-onset at the age of 6 months (152.4 ± 9.7 pg/ml; range: 124.9–191.2) compared to mock vector treated littermates (245.5 ± 13.4 pg/ml; range: 197.3–280.7; Fig. 3A; *p* < 0.05). This reduction of NF-L levels in the AAV9-*Mpz.GJB1* treatment group by 1.6-fold compared to the mock group is in accordance with their improved motor function indicating that NF-L levels can be used as a treatment-responsive and clinically relevant biomarker for future gene therapy.

In addition to NF-L, we evaluated NCAM-1, a novel blood biomarker with potential relevance to peripheral nerve disease. Similar to NF-L, analysis of NCAM-1 levels showed an elevation in untreated *Gjb1*-null mice (179787.4 ± 6953.2 nmol/μl; *n* = 4) compared to WT mice (135494.5 ± 10649.9 nmol/μl; *n* = 3; *p* > 0.05; Fig. 3B). Importantly, there was a significant amelioration of NCAM-1 elevation in the treated group (140651.6 ± 6798.4 nmol/μl; *n* = 7) compared to the mock-treated mice (196598 ± 9424.6 nmol/μl; *n* = 7; *p* < 0.001).

### Improved pathology in pre-onset AAV9-*Mpz.GJB1* treated *Gjb1*-null mice

**Fig. 4:**
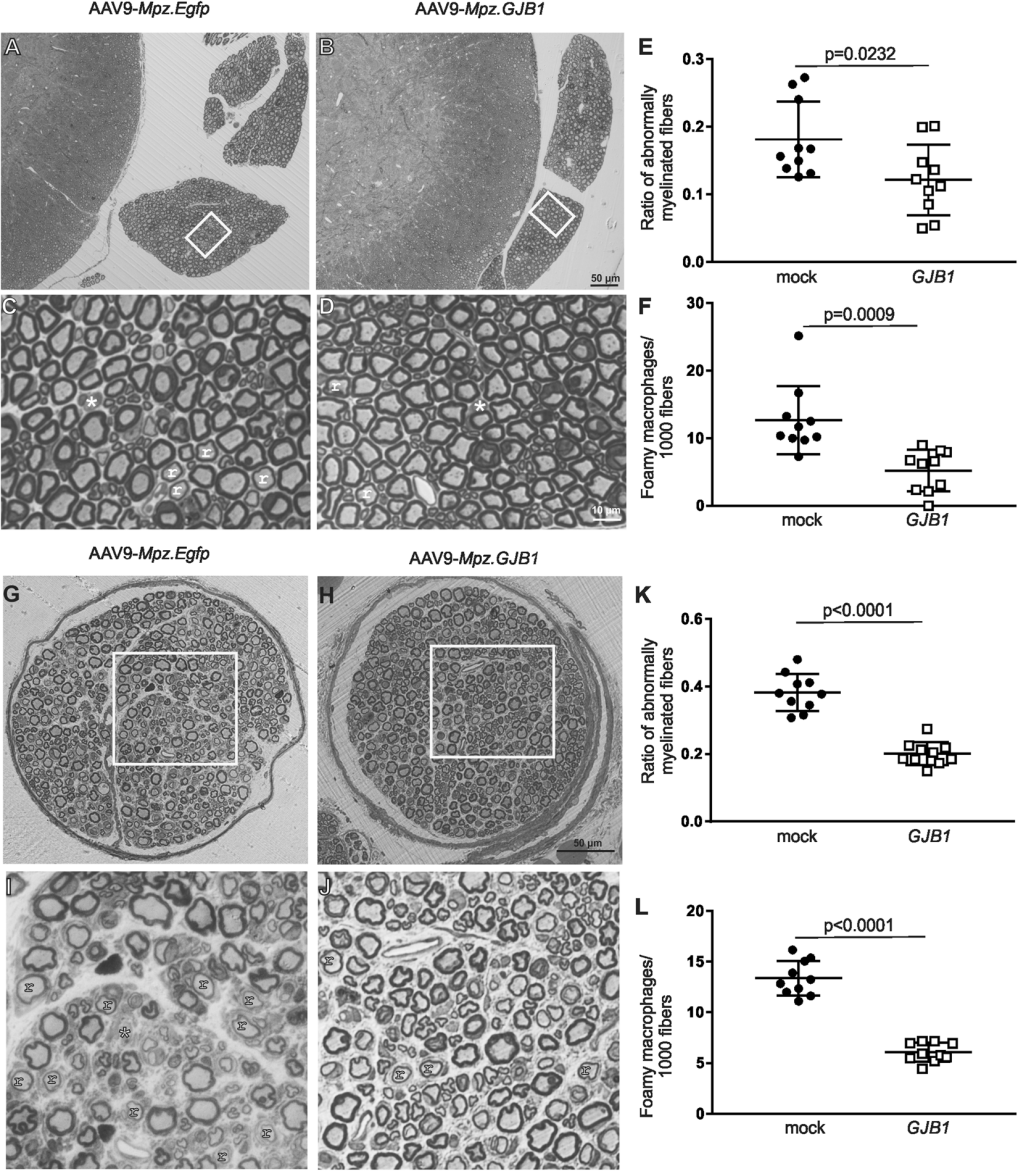
Morphological analysis of anterior lumbar roots and femoral motor nerves of *Gjb1*-null mice following pre-onset intrathecal delivery of the AAV9-Mpz.GJB1 vector. These are representative images of semithin sections of anterior lumbar spinal roots attached to the spinal cord at low (**A**) and (**B**) and higher (**C**) and (**D**) magnification, with morphometric analysis results (**E**) and (**F**), as well as of semithin sections of femoral motor nerves at low (**G**) and (**H**) and higher (**I**) and (**J**) magnification and corresponding morphometric analysis results (**K**) and (**L**), from mock and full vector treated mice as indicated, at 6 months of age (4 months after treatment). AAV9-*Mpz.GJB1* injected mice show improved myelination in all tissues compared to mock-treated littermates with fewer demyelinated (*) and remyelinated (*r*) fibers. Quantification of the ratios of abnormally myelinated fibers in multiple roots and femoral nerves (*n* = 10 mice per group) confirms significant improvement in the numbers of abnormally myelinated fibers (**E**) and (**K**), as well as reduction in the numbers of foamy macrophages (**F**) and (**L**) in the treated compared with mock treated littermates (see also Tables S1 and S3). Scale bars: (**A**, **B**), (**G**, **H**) 50 μm, (**C**, **D**), (**I**, **J**) 10 μm.

We performed a morphological analysis in transverse semithin sections of anterior lumbar roots, mid-sciatic and femoral motor nerves of 6-month-old *Gjb1*-null mice injected at the age of 2 months with either the full (AAV9.*Mpz-GJB1*) or the mock (AAV9.*Mpz-Egfp*) vector. Multiple roots, bilateral sciatic and femoral motor nerves from each mouse were examined and the ratio of abnormally myelinated fibers was counted and their proportion to the total number of fibers was calculated [32, 33, 42]. We also counted the number of foamy macrophages in each section [32], and calculated their numbers per 1,000 myelinated fibers (to account for variations in root and nerve size). In the anterior lumbar roots, a reduction of the abnormally myelinated fibers and foamy macrophages was observed in early AAV9-*Mpz.GJB1* treated mice compared to their mock-treated littermates (Fig. 4A–D). The ratio of abnormally myelinated fibers reached 0.12 ± 0.02 in the fully treated group compared to 0.18 ± 0.02 in the mock-treated mice (*n* = 10 mice per group; *p* = 0.0232; Fig. 4E and Table S1). Reduction was also observed in the number of foamy macrophages that reached 5.2 ± 0.97/1000 fibers in the treated group and 12.7 ± 1.60 in the mock group (*p* = 0.0009; Fig. 4F and Table S1).

Likewise, in femoral motor nerves, the ratio of abnormally myelinated fibers and the numbers of foamy macrophages were reduced after gene therapy (Fig. 4G–J). The ratio of abnormally myelinated fibers was 0.20 ± 0.01 in the treated group and 0.38 ± 0.02 in the mock group (*n* = 10 per group; *p* < 0.0001; Fig. 4K and Table S3), while foamy macrophages were reduced to 6.1 ± 0.30/1000 fibers in the treated group compared to 13.4 ± 0.54 in the mock group (*p* < 0.0001; Fig. 4L and Table S3). The sciatic nerves showed similar results (Supplementary results, Table S2 and Fig. S5A–F).

### Improved pathology in post-onset AAV9-*Mpz.GJB1-treated Gjb1*-null mice

**Fig. 5:**
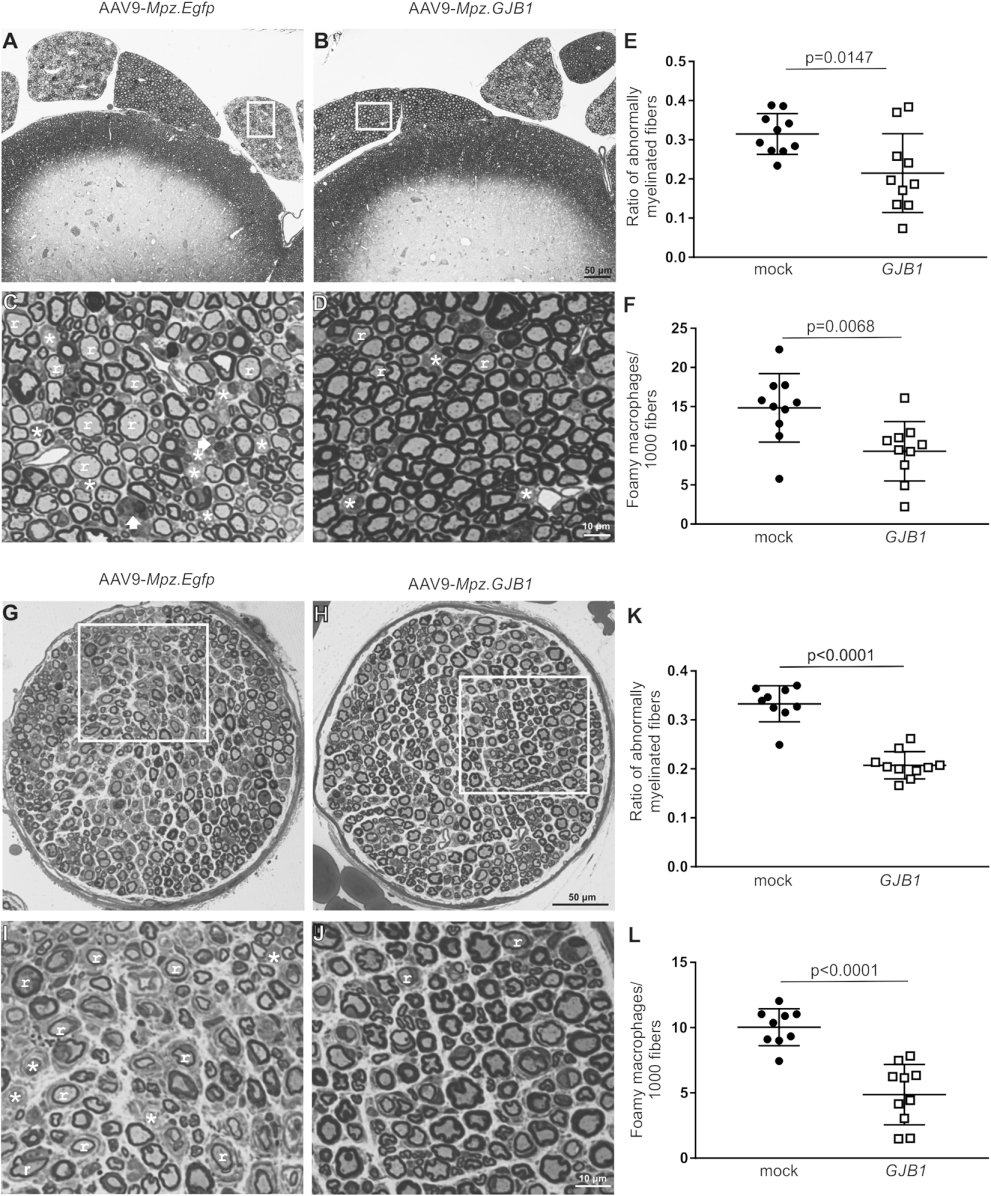
Morphological analysis of anterior lumbar roots and femoral motor nerves of *Gjb1*-null mice following post-onset intrathecal delivery of the AAV9-Mpz.GJB1 vector. These are representative images of semithin sections of anterior lumbar spinal roots attached to the spinal cord at low (**A**) and (**B**) and higher (**C**) and (**D**) magnification, with morphometric analysis results (**E**) and (**F**), as well as of semithin sections of femoral motor nerves at low (**G**) and (**H**) and higher (**I**) and (**J**) magnification with related morphometric analysis results (**K**) and (**L**), from mock and full (*GJB1*) vector treated mice as indicated, at 10 months of age (4 months after treatment). AAV9-*Mpz.GJB1* injected mice show improved myelination compared with mock-treated littermates with fewer demyelinated (*) and remyelinated (r) fibers in both tissues as well as fewer foamy macrophages (arrows in C). Quantification of the ratios of abnormally myelinated fibers in multiple roots and femoral nerves (*n* = 10 mice per group) confirms significant improvement in the numbers of abnormally myelinated fibers (**E**) and (**K**) as well as significant reduction in the numbers of foamy macrophages (**F, L**) in the treated compared with mock treated littermates (see also Tables S4 and S6). Scale bars: (**A**, **B**), (**G**, **H**) 50 μm, (**C**, **D**), (**I**, **J**) 10 μm.

As with early, pre-onset treatment groups, we performed a morphological analysis in transverse semithin sections of anterior lumbar roots, mid-sciatic, and femoral motor nerves of 10-month-old *Gjb1*-null mice injected either with the full or mock vector at the age of 6 months. Tissues of 10-month old WT and untreated *Gjb1*-null mice were also examined for assessment of the magnitude of the therapeutic effect. In anterior lumbar roots*,* similar to the early treated mice, post-onset treated mice showed improvement in morphological properties with a reduced ratio of abnormally myelinated fibers and numbers of foamy macrophages (Fig. 5A–D). The ratio of abnormally myelinated fibers decreased to 0.22 ± 0.03 in the treatment group (*n* = 10) compared to 0.32 ± 0.02 in the mock group (*n* = 10; *p* = 0.0147) (Fig. 5E and Table S4). Furthermore, the number of foamy macrophages was 9.31 ± 1.2/1000 fibers in the full group (*n* = 10) compared to 14.85 ± 1.38 in the mock group (*n* = 10; *p* = 0.0068) (Fig. 5F and Table S4). The sciatic nerves showed similar results (Supplementary results, Table S5 and Fig. S5G–L).

Finally, as in lumbar motor roots and sciatic nerves, we observed improved morphological properties also in the femoral motor nerves of post-onset treated mice (Fig. 5G–J), where the ratio of abnormal fibers reached 0.21 ± 0.009 in the treated group compared to 0.33 ± 0.012 in the mock group (*n* = 10 per group; *p* < 0.0001) (Fig. 5K and Table S6). Moreover, foamy macrophages were reduced to 4.9 ± 0.73/1000 fibers in the treated group (*n* = 10) compared to 10.0 ± 0.47 in the mock group (*n* = 10; *p* < 0.0001) (Fig. 5L and Table S6). Thus, our morphological analysis of the PNS tissues revealed a significant improvement of the pathology including de- and remyelination and inflammatory changes that are characteristic of this model of CMT1X both in pre- as well as in post-onset treated mice. However, morphological improvement in treated mice was only partial, as demonstrated by the comparison to WT tissues (Tables S4–S6). On the other hand, comparison of 10-month old mock treated with untreated *Gjb1*-null mice of the same age showed similar degree of pathology (Supplementary results, Fig. S6, and Tables S4–6), indicating that mock vector injection and EGFP expression had no detrimental effects in this model.

## Discussion

In this study, we used an AAV9 vector for targeted gene delivery to Schwann cells in order to treat the peripheral neuropathy in the *Gjb1*-null mouse model of CMT1X. Using the *GJB1* gene coding sequence driven by the rat *Mpz* promoter with known specificity for myelinating Schwann cells [27, 32] we delivered by a single lumbar intrathecal injection the AAV9 vector into 2- and 6-month-old *Gjb1*-null mice and achieved widespread expression of the virally delivered human Cx32. This treatment resulted in significant improvements in all outcome measures in this neuropathy model both when treated at early as well as at later stages of the disease.

AAV vectors are known to provide high expression levels in transduced cells [55–57] offering the potential to treat patients with different CMT1X mutations, including those with potential dominant-negative effects [33]. Moreover, the use of AAV vector provides a more translatable approach since these vectors remain mostly episomal minimizing the risk for insertional mutagenesis [58]. In contrast, lentiviral vectors integrate into the host genome [35] increasing the risk for mutagenesis and therefore have limited potential for systemic in vivo delivery. Importantly, the AAV9 vector is already used in clinical trials with an established safety profile and recently FDA-approved application for the treatment of spinal muscular atrophy.

Intrathecal injection of the AAV9 vector resulted in widespread expression of the reporter gene EGFP in WT mice as well as of Cx32 in both 2- and 6-month-old *Gjb1*-null mice. Analysis of vector genomes in PNS tissues indicates a gradient of biodistribution from proximal to distal PNS tissues, although there was considerable variability between animals. As expected, muscle and spinal cord transduction by the AAV vectors were also evident, but EGFP or Cx32 expression was restricted to myelinating Schwann cells under the control of *Mpz* promoter. Moreover, we demonstrate that virally delivered Cx32 is correctly localized in the paranodal areas of myelinating Schwann cells as previously achieved with lentiviral gene delivery [31, 32]. However, AAV9 appears to provide higher expression rates and levels as evident by the immunoblot analysis in PNS tissues of injected mice. In this study, we injected AAV amounts of 2 × 10^10^ vg. Higher vector titers allowing the injection of more viral particles exceeding >10^11^ vg may improve further the biodistribution into the PNS and expression rates. Furthermore, it remains to be shown whether the biodistribution achieved by intrathecal injection can be also obtained in a larger animal to support the clinical application.

Although AAV9 provided higher Cx32 expression levels compared to our previous studies, only a subset of Schwann cells expressed the gene of interest resulting only in partial phenotype correction. Interestingly, it appears that restoring Cx32 function in at least 50% of myelinating Schwann cells in PNS tissues may be sufficient to provide significant functional and morphological improvement in this model and potentially also in CMT1X patients. This partial correction may reflect the situation caused by the random X chromosome inactivation in female patients affected by CMT1X, who typically present with a milder and non-progressive phenotype [59]. As thousands of myelinating Schwann cells are needed to myelinate the length of a single axon, there may be a threshold of functioning Schwann cells ratio for maintaining axon survival explaining why the correction of a subset of Schwann cells was sufficient to improve the outcome in the disease model.

In this study, we demonstrate that targeted transduction of Schwann cell of the peripheral nervous system can be achieved after a single lumbar intrathecal injection using the AAV9. AAV9 has been previously used mostly for CNS transduction [60] but has not been studied specifically for PNS targeting [61–63]. However, some studies showed that AAV9 can transduce peripheral nerves after intrathecal injection [64] with detection of EGFP expression in sciatic nerves after intravenous or intrathecal injection in mice [65, 66]. AAV9 has been mainly used in experimental models of giant axonal neuropathy [61], spinal muscular atrophy [67], and amyotrophic lateral sclerosis [68, 69]. These studies demonstrated that AAV9 can transduce efficiently the CNS resulting in phenotype rescue of these models. Furthermore, the AAV9 vector has been shown to provide gene expression in non-human primates either by intravenous [70] or by intrathecal [71] and recently by subpial delivery [68]. However, there are limited data concerning the efficacy of AAV9 to transduce the PNS. A recent study showed a gradient of AAV9 biodistribution into the PNS following lumbar intrathecal injection or injection in the cisterna magna, with reporter gene expression also in the sciatic nerves [64]. In contrast to our study, cell specificity of expression could not be demonstrated, since ubiquitous promoters were used. The lower efficacy of Schwann cell transduction after intrathecal injection in other studies [64] could be in part attributed to the fourfold lower volumes injected compared to our approach.

Improvement of functional properties in AAV9-*Mpz.GJB1* treated groups, including behavioral and electrophysiological outcomes, indicate that treatment with this vector not only results in expression of human Cx32 but also in restoration of Cx32 function in myelinated fibers. Although pre- and post-onset treatment groups were studied at different time periods and may not be directly comparable, almost similar improvements in functional properties were observed at both time points, indicating that gene replacement can be beneficial either before or after the onset of the neuropathy, reversing some of the pathological changes that are already present in older mice. Early treated mice showed a trend for higher increase of hind limb force and more improvement of MNCVs approaching those of age-matched WT mice, while in post-onset treated mice they remained significantly below those of WT mice. Thus, early intervention may provide a bigger therapeutic benefit, although not clearly demonstrated in all outcome measures, in accordance with our previous studies [31, 33]. The relatively slow progression of the pathological and functional changes beyond 6 months of age in the *Gjb1*-null model at baseline [25, 31, 33] may account for these observations.

By the time CMT1X patients become symptomatic, typically by late childhood to young adulthood, they are likely to have advanced pathological changes in their nerves including demyelination and progressive axonal loss [5, 23, 24, 72, 73]. Although the *Gjb1*-null mouse model does not fully reproduce the degree of pathology in patients, it develops a significant degree of demyelination and axonal pathology especially in motor fibers at the ages of 6 to 10 months [25, 26, 30, 31, 52] in addition to inflammatory changes with macrophage infiltration [53, 54]. Thus, demonstration of post-onset efficacy of gene therapy in this model indicates that viral expression and restoration of Cx32 function can be effective even in an intraneural environment of ongoing chronic de- and re-myelination, axonal degeneration, and inflammation, and is therefore highly relevant for the potential of clinical application.

The therapeutic benefit in this neuropathy model is further demonstrated by the amelioration of NF-L plasma concentrations in AAV9-*Mpz.GJB1* treated compared to the mock-treated mice. NF-L is a biomarker that correlates with peripheral nerve degeneration in patients with CMT1X and other CMT types [51]. Since NF-L is associated with axonal damage, we conclude that treatment with AAV9-*Mpz.GJB1* either prevents or reverses axonal damage caused by the loss of Cx32 in Schwann cells. Similar treatment responsiveness of this biomarker was shown in our previous studies using lentiviral gene therapy in CMT1X and CMT4C demyelinating neuropathy models [31, 74]. Interestingly, in this study AAV9-treated *Gjb1*-null mice showed an even stronger amelioration of NF-L levels approaching those of WT mice, indicating that the therapeutic benefit and axonal protection provided by AAV are stronger. These findings support the use of the AAV9 vector for the treatment of demyelinating neuropathies and confirm NF-L as a relevant biomarker that can be used to measure treatment response in CMT1X patients.

A further blood biomarker reflecting nerve pathology, NCAM1, also showed significant treatment responsiveness. Elevated NCAM1 serum levels have been previously associated with various forms of inflammatory neuropathies and with CMT1A [75] as well as with Alzheimer’s disease [76], indicating that it may be a relatively broad marker of CNS and PNS neurodegeneration. Furthermore, NCAM-1 was shown to regulate synaptic reorganization after peripheral nerve injury suggesting an important role during regeneration [77]. We demonstrate here for the first time a significant increase of NCAM-1 levels in 10-month-old *Gjb1*-null mice compared to WT controls, highlighting its relevance for CMT1X pathology. Importantly, treated *Gjb1*-null mice showed a significant amelioration in serum NCAM-1 levels compared to the mock group at 10 months of age, suggesting the potential value of this biomarker in CMT1X. However, further confirmatory studies in the serum of CMTX1 patients are required to validate this finding.

Our morphological analysis revealed that treatment with the AAV9 vector results in improvement of the pathological properties of all the PNS tissues examined. Both the ratios of abnormally myelinated fibers and the numbers of foamy macrophages were reduced in fully treated mice compared to the mock group in both pre- and post-onset studies. The percentage of improvement reached 60% for the roots and the femoral nerve and 50% for the sciatic nerves, although baseline values were higher in late treated mice as expected based on the pathology progression in the *Gjb1*-null mouse model [30]. Stronger improvement in tissues with motor fibers including the anterior lumbar roots and femoral motor branches likely reflects the fact that neuropathy in this model affects predominantly the motor fibers [25, 78]. Overall, our data indicate that AAV9-mediated delivery of Cx32 can improve PNS pathology both before and after the onset of the CMT1X neuropathy.

In conclusion, this study provides the proof of principle that intrathecal injection of AAV9, a well-established vector for clinical applications, provides widespread biodistribution in the PNS and efficient transduction of myelinating Schwann cells. Moreover, Schwann cell-targeted expression of Cx32 driven by a myelin-specific promoter can prevent or even reverse the demyelination and axonal degeneration occurring in the CMT1X mouse model providing a long-lasting therapeutic benefit even after the onset of the neuropathy. Evaluation of this approach in larger animal models would be valuable in order to confirm adequate biodistribution and safety facilitating the path towards clinical translation for treating CMT1X patients.

## Supplementary information

Supplementary material
