# Supplementary material for: AAV9-mediated Schwann cell-targeted gene therapy rescues a model of demyelinating neuropathy
Source: Gene Ther. 2021 Mar 10;28(10-11):659–75. doi: 10.1038/s41434-021-00250-0 (PMC8599011; doi:10.1038/s41434-021-00250-0)
Supplement: Supplementary file 1 — Supplementary material [file 41434_2021_250_MOESM1_ESM.pdf]

SUPPLEMENTARY MATERIAL

1. Supplementary Results
2. Supplementary Tables S1-6
3. Supplementary Figures S1-6

1. Supplementary Results

Morphometric analysis of mid-sciatic nerves

In sciatic nerves of pre-onset treated mice, a reduction of the ratio of abnormally myelinated fibers and of the numbers of foamy macrophages was also observed (**Fig. S5A-D)**. The ratio of abnormally myelinated fibers reached 0.05±0.003 in the AAV9.*Mpz-GJB1* treated mice and 0.12±0.004 in the mock treated mice (n=10 per group; p<0.0001; **Fig. S5E and Table S2**). Moreover, the numbers of foamy macrophages decreased to 4.6±0.60/1000 fibers in the treated group compared to 8.1±0.66 in the mock group (p=0.001; **Fig. S5F and Table S2**).

In the sciatic nerves of post-onset treatment groups, the ratio of abnormal fibers decreased to 0.06±0.003 in the treated group compared to 0.11±0.004 in the mock (n=10 per group; p<0.0001) (**Fig. S5G-K and Table S5**). Foamy macrophages were also reduced to 3.8±0.69/1000 fibers in the treated compared to 7.8±0.48 in the mock group (n=10 per group; p=0.0005) (**Fig. S5L and Table S5**).

Morphometric analysis of PNS tissues of untreated *Gjb1*-null compared to AAV9-Mpz.*Egfp* treated *Gjb1*-null mice

Furthermore, we compared *Gjb1*-null untreated and *Gjb1*-null AAV9-Mpz.*Egfp* treated mice in order to assess the effects of the injection and to exclude any toxic effects of the reporter gene expression. At 10 months of age, untreated mice (n=7 mice for anterior roots and n=4 for femoral nerves) had similar numbers of abnormally myelinated fibers and foamy macrophages compared to the mock treated mice (n=10). The numbers of abnormally myelinated fibers reached 0.38±0.031 in anterior lumbar roots while foamy macrophages reached 18.23±1.10 in the untreated group while the numbers in the mock-treated reached 0.32±0.016 ($p>0.05$) and 14.85±1.38 ($p>0.05$), respectively (**Table S4**). Similar to the lumbar roots, in the sciatic (**Table S5)** and femoral (**Table S6)** nerves the ratio of abnormally myelinated fibers reached 0.10±0.018 and 0.33±0.04, respectively, in the untreated group compared to 0.11±0.004 ($p>0.05$) and 0.33±0.01 ($p>0.05$), respectively. in the mock group. Foamy macrophages reached 6.89±1.25 ($p>0.05$;) in sciatic nerves and 7.85±2.08 in femoral nerves of the untreated group while the values for the mock group reached 7.76±0.48 ($p>0.05$) and 10.03±0.47 ($p>0.05$), respectively.

2. Supplementary Tables

Table S1: Results of morphometric analysis of anterior lumbar roots in *pre-onset* treated *Gjb1*-null mice at 6 months of age. Values represent mean ± SEM.

	**AAV9-*Mpz.Egfp* (mock) injected**	**AAV9-*Mpz.GJB1* (full) injected**	**Mann-Whitney test**
**Anterior lumbar roots**	(total 43 roots from n=10 mice)	(total 43 roots from n=10 mice)	
**Total fibers counted**	10605	8232	
**Ratio abnormally myelinated fibers**	0.18± 0.056	0.12 ± 0.052	p=0.0232
**Number of macrophages/ 1000 fibers**	12.68 ± 5.05	5.22 ± 3.07	p=0.0009

Table S2: Results of morphometric analysis of sciatic nerves in *pre-onset* treated *Gjb1*-null mice at 6 months of age. Values represent mean ± SEM.

	**AAV9-*Mpz.Egfp* (mock) injected**	**AAV9-*Mpz.GJB1* (full) injected**	**Mann-Whitney test**
**Sciatic nerves**	(total 20 nerves from n=10 mice)	(total 20 nerves from n=10 mice)	
**Total fibers counted**	33970	37822	
**Ratio abnormally myelinated fibers**	0.12 ± 0.004	0.05± 0.003	p<0.0001
**Number of macrophages/ 1000 fibers**	8.07±0.66	4.56±0.60	p=0.001

Table S3: Results of morphometric analysis of femoral motor nerves in *pre-onset* treated *Gjb1*-null mice at 6 months of age. Values represent mean ± SEM.

	**AAV9-*Mpz.Egfp* (mock) injected**	**AAV9-*Mpz.GJB1* (full) injected**	**Mann-Whitney test**
**Femoral nerves**	(total 20 nerves from n=10 mice)	(total 20 nerves from n=10 mice)	
**Total fibers counted**	6631	6346	
**Ratio abnormally myelinated fibers**	0.39±0.02	0.20±0.01	p<0.0001
**Number of macrophages/ 1000 fibers**	13.37±0.54	6.06±0.30	p<0.0001

Table S4: Results of morphometric analysis of anterior lumbar roots in *post-onset* treated *Gjb1*-null mice at 10 months of age. Values represent mean ± SEM. Results of age-matched wild type (WT) and untreated *Gjb1*-null mice are also included for comparison.

	**AAV9-*Mpz.Egfp* (mock) injected *Gjb1*-null**	**AAV9-*Mpz.GJB1* (full) injected *Gjb1*-null**	**WT**	***Gjb1*-null untreated**	**Mann-Whitney test (mock vs full)**
**Anterior lumbar roots**	(total 39 roots from n=10 mice)	(total 36 roots from n=10 mice)	(total 15 roots from n=4 mice)	(total 21 roots from n=7 mice)	
**Total fibers counted**	9144	10082	3745	4997	
**Ratio abnormally myelinated fibers**	0.32±0.016	0.22±0.032	0.005±0.001	0.38±0.031	p=0.0147
**Number of macrophages/ 1000 fibers**	14.85±1.38	9.31±1.20	0.25±0.25	18.23±1.10	p=0.0068

Table S5: Results of morphometric analysis of sciatic nerves in post-onset treated *Gjb1*-null mice at 10 months of age. Values represent mean ± SEM. Results of age-matched wild type (WT) and untreated *Gjb1*-null mice are also included for comparison.

	**AAV9-*Mpz.Egfp* (mock) injected *Gjb1*-null**	**AAV9-*Mpz.GJB1* (full) injected *Gjb1*-null**	**WT**	***Gjb1*-null untreated**	**Mann-Whitney test (mock vs full)**
**Sciatic nerves**	(total 20 nerves from n=10 mice)	(total 20 nerves from n=10 mice)	(total 4 nerves from n=4 mice)	(total 14 nerves from n=7 mice)	
**Total fibers counted**	35118	35134	7187	21231	
**Ratio abnormally myelinated fibers**	0.11±0.004	0.06± 0.003	0.002±0.0005	0.10±0.018	p<0.0001
**Number of macrophages/ 1000 fibers**	7.76±0.48	3.84±0.69	0.75±0.25	6.89±1.25	p=0.0005

Table S6: Results of morphometric analysis of femoral motor nerves in post-onset treated *Gjb1*-null mice at 10 months of age. Values represent mean ± SEM. Results of age-matched wild type (WT) and untreated *Gjb1*-null mice are also included for comparison.

	**AAV9-*Mpz.Egfp* (mock) injected *Gjb1*-null**	**AAV9-*Mpz.GJB1* (full) injected *Gjb1*-null**	**WT**	***Gjb1*-null untreated**	**Mann-Whitney test (mock vs full)**
**Femoral nerves**	(total 20 nerves from n=10 mice)	(total 20 nerves from n=10 mice)	(total 4 nerves from n=4 mice)	(total 8 nerves from n=4 mice)	
**Total fibers counted**	5559	6581	1284	2434	
**Ratio abnormally myelinated fibers**	0.33±0.01	0.21±0.01	0.004±0.0008	0.33±0.04	p<0.0001
**Number of macrophages/ 1000 fibers**	10.03±0.47	4.87±0.73	0.50±0.29	7.85±2.08	p<0.0001

2. Supplementary Figures

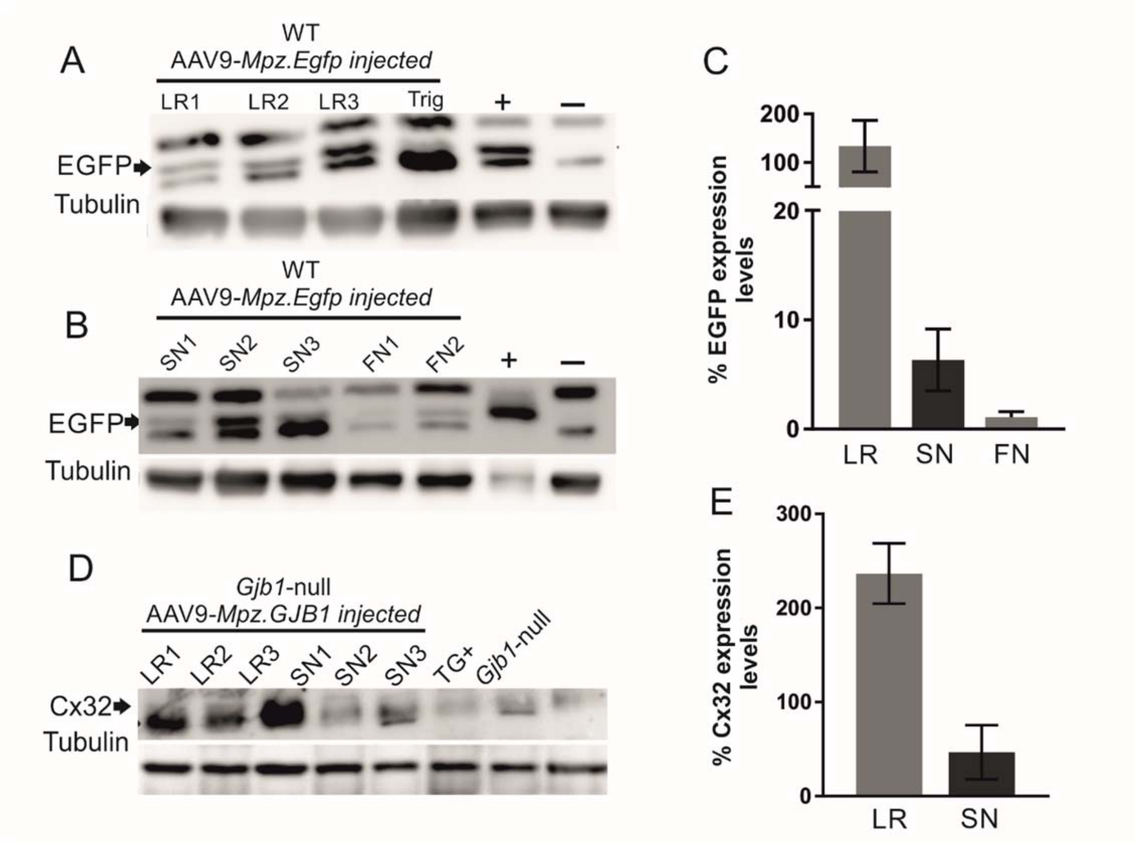

Figure S1: EGFP and Cx32 expression levels in AAV injected WT and *Gjb1*-null mice. **A-B**: Immunoblot analysis of lumbar roots (LR), sciatic nerves (SN) and femoral nerves (FN) as well as of a trigeminal nerve sample (Trig) from AAV9-Mpz.*Egfp* injected WT mice (n=3) confirms the presence of the specific EGFP band as opposed to a non-injected mouse nerve run as negative control (−). Positive control root sample (+) is from a mouse transgenically expressing EGFP. β-Tubulin serves as a loading control. **C:** Quantification of the EGFP expression levels normalized for tubulin levels in PNS tissues from injected mice relative to the positive control shows a gradient with expression levels in lumbar roots reaching 133.58±52.93 %, in sciatic nerves 6.33±2.84 % and in femoral nerves 1.08±0.51 % of the positive control root sample. **D:** Immunoblot analysis of different tissues from AAV9-Mpz.*GJB1* injected *Gjb1*-null mice as indicated, as opposed to a non-injected littermate mouse run as negative control (−) confirms Cx32 expression. TG+ is a positive control root

sample from a mouse transgenically expressing Cx32. **E:** Quantification of the Cx32 expression levels normalized for tubulin loading relative to the positive control shows Cx32 levels of 236.73±32.08 % in lumbar roots and 46.63±28.80 % in sciatic nerves. Values in C and E represent Mean±SEM

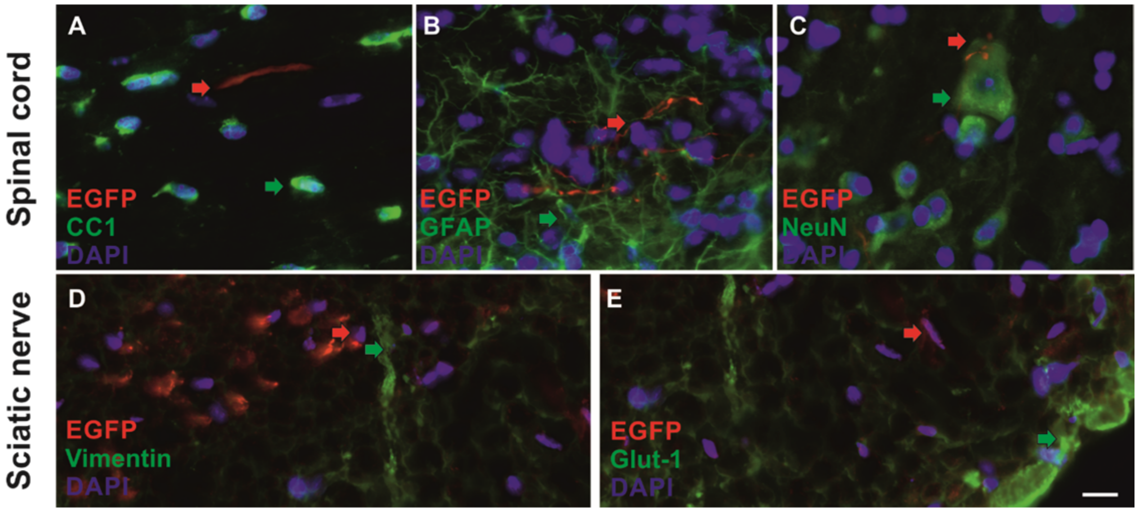

Figure S2: Specificity of vector expression after intrathecal delivery of AAV9-*Mpz.Egfp* in WT mice. A-C: Immunofluorescence staining for EGFP (red) and various cell markers (green) of spinal cord sections from AAV9-*Mpz.Egfp* injected WT mice does not show any expression of EGFP in CC1-positive oligodendrocytes (green arrows) (**A**), in GFAP-positive astrocytes (**B**), or in NeuN-positive neurons (**C**). In some central fibers there is evidence of EGFP immunoreactivity (red arrows) likely representing EGFP transported from peripheral axons into the CNS, since no EGFP was detected in the perikarya of CNS cells. **D-E:** Immunofluorescence staining of sciatic nerve section also showed that EGFP was expressed in perinuclear cytoplasm of myelinating Schwann cells (red arrows) but not in vimentin-positive fibroblasts (**D**) or in Glut-1 positive perineurial cells (**E**). Scale bar: 20 µm.

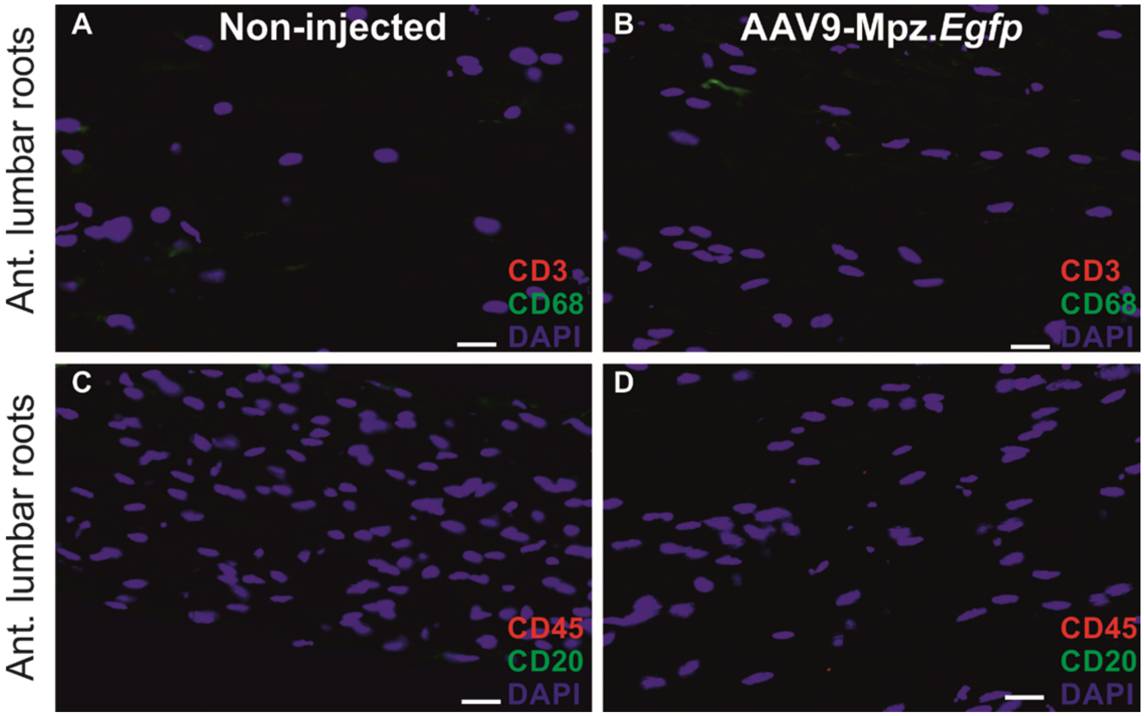

Figure S3: Lack of immune reaction after intrathecal delivery of AAV9-*Mpz.Egfp* in WT mice. Anterior lumbar roots of AAV9-*Mpz.Egfp* injected WT mice (**B**) do not show any significant immune response compared to the non-injected WT mice tissues (**A**) as indicated by double immunostaining with T-cell marker CD3 (red) and macrophage marker CD68 (green). Further staining with B-cell marker CD20 (green) and leukocyte marker CD45 (red) also shows no presence of immune cells in the tissues of injected (**D**) compared to non-injected mice (**C**). Cell nuclei are stained with DAPI (blue). Scale bar: 20 μm.

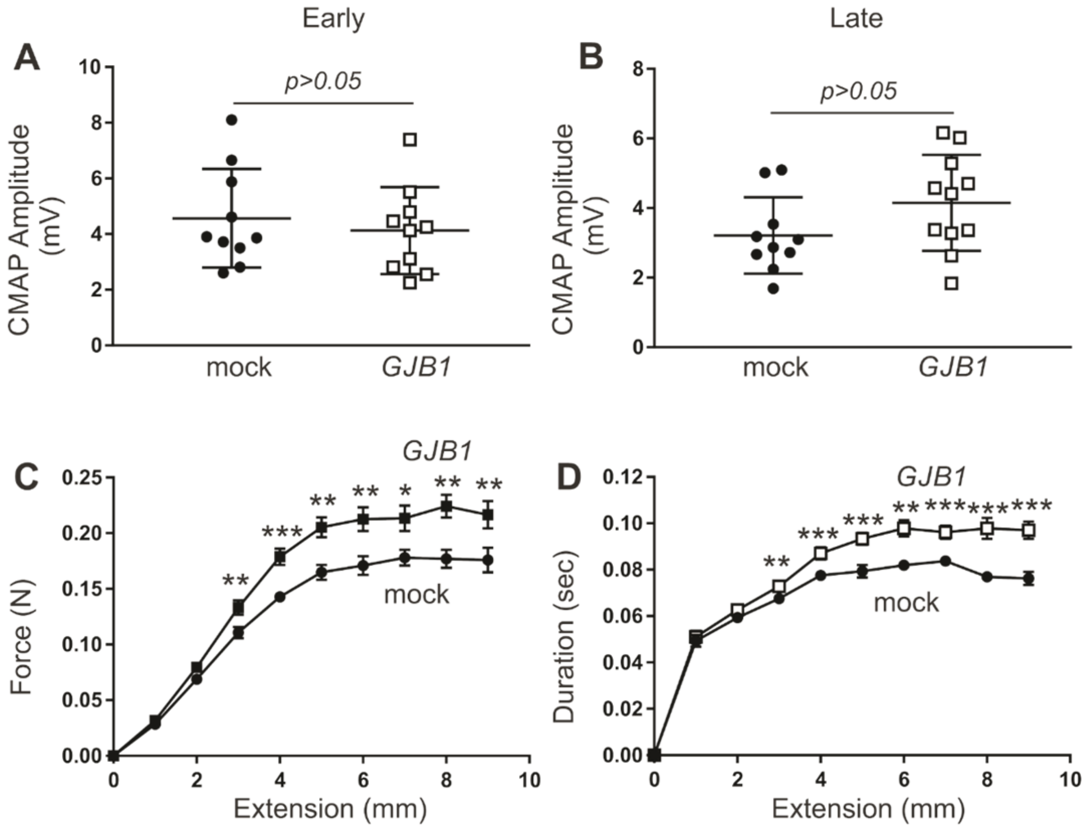

Figure S4: Electrophysiological analysis of pre- and post-onset AAV9-*Mpz.GJB1* injected *Gjb1*-null mice compared to mock treated littermates. Electrophysiological analysis of sciatic nerve motor responses did not show any differences in the amplitude of the compound muscle action potential (CMAP) in pre-onset (**A**) or post-onset (**B**) treated groups. **C-D**: The results of quadriceps muscle contractility in post-onset treated and mock-treated groups show increased force (**C)** and duration (**D**) of the muscle contraction in treated (*GJB1*; *n*=10) mice at different extensions (indicated in millimetres) compared to the mock-treated mice (mock; *n*=10) at 10 months of age (*:p<0.05;**:p<0.01; ***:p<0.001). Values in C and D represent Mean±SEM

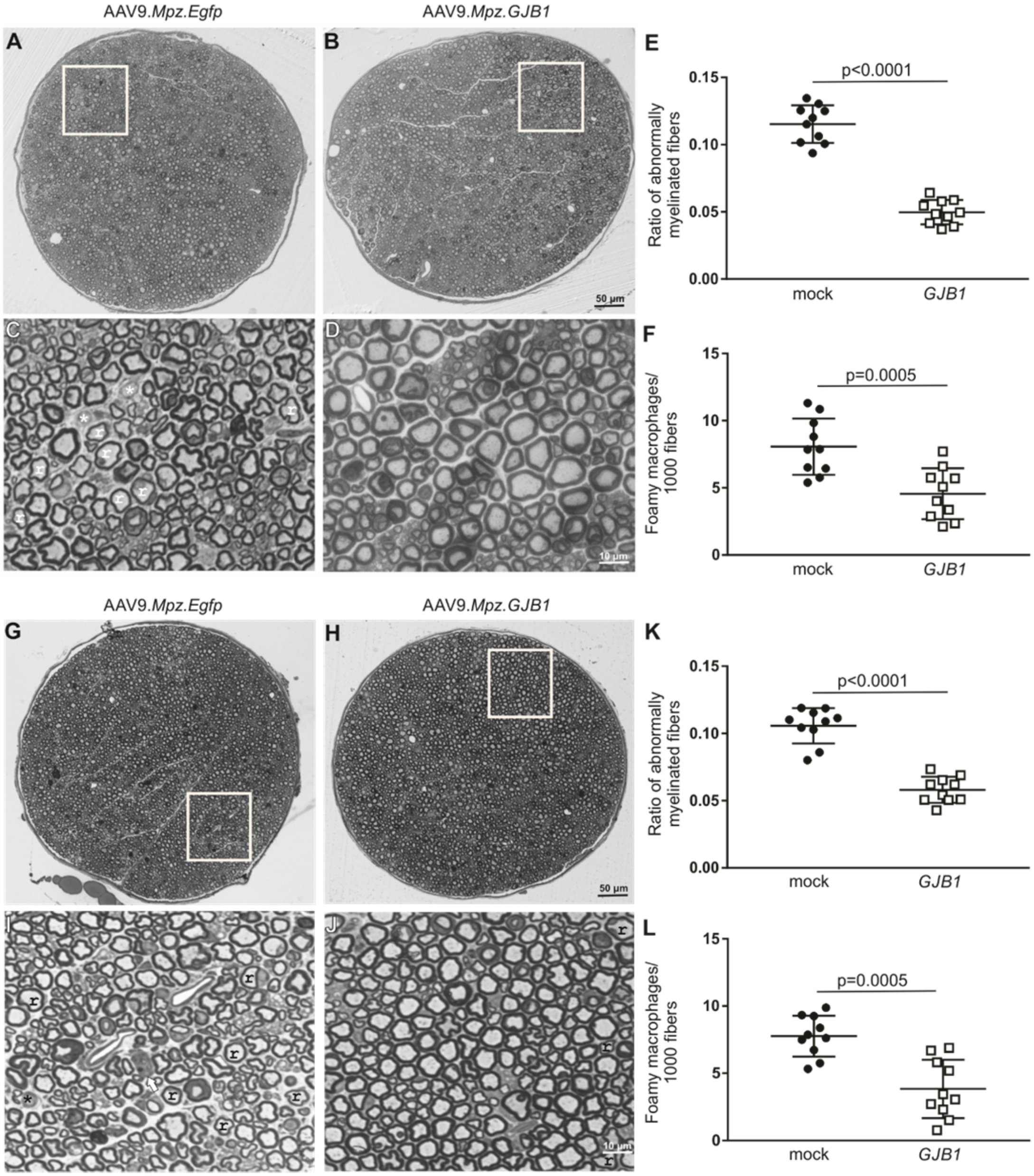

Figure S5: Morphological analysis of *Gjb1*-null mice sciatic nerves following pre- and post-onset intrathecal delivery of the AAV9-*Mpz.GJB1* or mock vector. These are representative images of semithin sections of sciatic nerves of pre-onset treated mice at low (**A-B**) and higher (**C-D**) magnification, with morphometric analysis results (**E-F**), as well as of post-onset treated mice at low (**G-H**) and higher (**I-J**) magnification, with morphometric analysis results (**K-L**) from mock and full (*GJB1*) vector treated mice as indicated, all at 4 months after treatment (at age 6 months for pre-onset and at age 10 months for post-onset

groups). AAV9-*Mpz.GJB1* injected mice show improved myelination compared with mock-treated littermates with fewer demyelinated (*) and remyelinated (r) fibers, as well as reduced numbers of foamy macrophages (white arrow in I). Quantification of the ratios of abnormally myelinated fibers in sciatic nerves (n = 10 mice per group) confirms significant improvement in the numbers of abnormally myelinated fibers (**E, K**), as well as significant reduction in the numbers of foamy macrophages (**F, L**) in treated compared with mock treated littermates at both treatment time points. Scale bars: A-B, G-H 50 μm, C-D, I-J 10 μm.

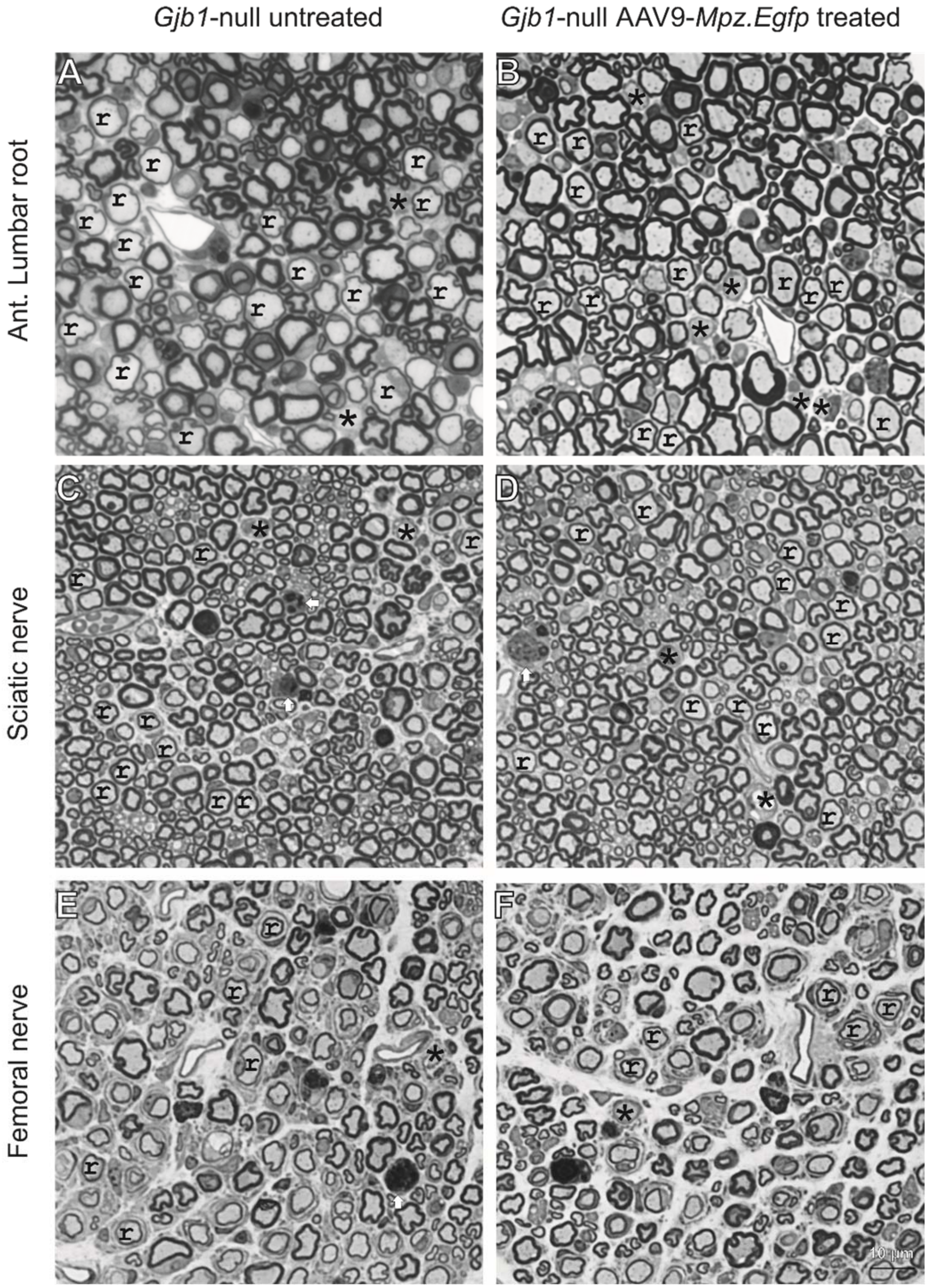

Figure S6: Semithin sections of different PNS tissues of untreated and mock-treated *Gjb1*-null mice (*Gjb1*-null AAV9-Mpz.*Egfp*). These are representative images of semithin sections of anterior lumbar roots (**A-B**), sciatic (**C-D**) and femoral nerves (**E-F**) from 10-

month old *Gjb1*-null mice that were either untreated (A, C, E) or mock-treated (B, D, F), as indicated. The degree of pathology is similar in both groups including the abnormally myelinated fibers (r), demyelinated fibers (*), as well as foamy macrophages (arrows). Quantification results are presented in Tables S4-6. Scale bar: 10 μm.
